# Recurrent mitral regurgitation after mitral valve repair for bileaflet lesions in the modern era

**DOI:** 10.1186/s13019-019-1035-3

**Published:** 2019-11-27

**Authors:** Daisuke Kaneyuki, Hiroyuki Nakajima, Toshihisa Asakura, Akihiro Yoshitake, Chiho Tokunaga, Masato Tochii, Jun Hayashi, Akitoshi Takazawa, Hiroaki Izumida, Atsushi Iguchi

**Affiliations:** Division of Cardiovascular Surgery, Saitama Medical University International Medical Center, 1397-1, Yamane, Hidaka-shi, Saitama, 350-1298 Japan

**Keywords:** Mitral valve repair, Bileaflet prolapse, Mitral regurgitation

## Abstract

**Background:**

Good mid-term durability of mitral valve repair of bileaflet lesions has been reported; however, patients may develop failure during follow-up. This study assessed late outcomes and mechanisms of failure associated with mitral valve repair of bileaflet lesions.

**Methods:**

Fifty-six patients (mean age 67 ± 12 years) underwent mitral valve repair of bileaflet lesions due to degenerative disease in 2011–2018. Mitral annuloplasty was added to all procedures except for 1 patient with annular calcification. Mitral valve lesions were identified by surgical inspection. Mean clinical and echocardiography follow-up occurred at 2.7 ± 2.1 and 2.5 ± 1.9 years, respectively.

**Results:**

Additional mitral valve repair techniques involved triangular resection (*n* = 15 patients), quadrangular resection with sliding plasty (*n* = 12), neochordoplasty (*n* = 52), and commissural plication (*n* = 26). Prolapse of ≥2 anterior and posterior leaflet scallops occurred in 22 (39%) and 30 (54%) patients, respectively. During follow-up, 10 (17.8%) patients developed moderate or severe mitral regurgitation. Whereas prolapse or tethering was observed early after neochordoplasty or quadrangular resection, recurrent regurgitation occurred late after commissural repair. Five-year freedom from recurrent moderate or severe mitral regurgitation rates was 71.1 ± 11.0%.

**Conclusions:**

Seventeen percent of patients developed recurrent mitral regurgitation during follow-up. Repair failure in the early phase occurred owing to aggressive resection of the posterior mitral leaflet or maladjustment of the artificial neochordae. Recurrent mitral regurgitation might occur in the late phase even after acceptable commissural repair. A sequential approach may be useful to improve the quality of mitral valve repair in bileaflet lesions.

## Background

Compared with promising long-term outcomes of mitral valve (MV) repair of isolated posterior leaflet lesions, anterior leaflet and bileaflet lesions have been recognized as a risk factor associated with repair failure [1–6]. Better outcomes of MV repair of anterior leaflet lesions have been reported after the emergence of several refined techniques, including neochordoplasty [2, 7, 8]. Despite the limited literature available, the long-term durability of MV repair of isolated commissural lesions is comparable to that of posterior leaflet lesions [9, 10]. Even repair of bileaflet lesions can be feasible and result in good mid-term durability when performed at a reference center [7, 11, 12]. However, some patients still develop recurrent mitral regurgitation (MR) after successful MV repair during the long-term follow-up period. A few studies have focused on late outcomes and mechanisms of failure associated with MV repair of bileaflet lesions requiring multiple reconstructive techniques [13, 14]. The objective of this study was to elucidate the mid-term outcomes and mechanisms of recurrent MR after MV repair of bileaflet lesions.

## Methods

### Patient population and follow-up

From 2011 to 2018, 575 patients underwent an MV procedure for severe MR at the Saitama Medical University International Medical Center, Hidaka-shi, Japan. Patients with an etiology of MR other than degeneration (ischemic, rheumatic, endocarditis, functional, or congenital) were excluded. Of the 351 patients subjected to the MV procedure for degenerative disease, 22 patients underwent MV replacement (93.7% repair rate). MV lesions were mainly determined by surgical inspection. Prolapse was defined as an override of the free edge of both leaflets above the annular plane during ventricular systole. Patients with a limited lesion of a commissure (< 5 mm) were not classified as having bileaflet lesions. After exclusion, 56 patients with bilateral lesions who underwent MV repair were eligible for this study (56/329, 16.0%).

Patients were followed up by our department or the referring cardiologist yearly after discharge. MR was initially classified as none, trivial, mild, moderate, and severe based on the length and area of the regurgitant jet. In addition to transesophageal echocardiography during the operation, every patient underwent a transthoracic study before hospital discharge and multiple studies thereafter. Follow-up information was collected through a mailed questionnaire or by telephone interview. Late echocardiographic studies performed by referring cardiologists were reviewed. The cause of death was determined by hospital charts review, death certificates, or information from the physician who was caring for the patient at that time. Clinical follow-up was for 2.7 ± 2.1 years. Postoperative echocardiography was completed for all patients and late echocardiography results were available for 93% of the patients. Mean echocardiography follow-up time was 2.5 ± 1.9 years (range, 29–2541 days).

### Operative technique

All surgeries were performed through a full median sternotomy in 51 patients (91%) or a small right anterolateral thoracotomy in 5 patients (9%). Valve repairs were performed by means of multiple reconstructive techniques, depending on the valve findings. Repair of posterior prolapsed leaflets was accomplished with resection (triangular in 15 [27%] and quadrangular with sliding plasty in 12 [21%]) and reconstruction with leaflet reapproximation, with or without artificial neochordae (Table 1). Artificial neochordae with 4–0 or 5–0 polytetrafluoroethylene sutures (Gore-Tex sutures, W. L. Gore & Associates, Newark, DE) were used preferentially for anterior prolapsed leaflets. In this cohort, artificial neochordae placement was performed in 52 (93%) patients. Commissural prolapse was repaired by means of commissural plication (26 patients, 52%) or reconstruction with artificial neochordae (2 patients, 4%) according to the size of the prolapse lesions. Notably, 24 patients who received leaflet resection also received artificial neochordal replacement.

**Table 1:** Patient Characteristics

Covariate	Mean ± SD or No. (%)
Demographics	
Age, years	67 ± 12
Female sex	19 (34)
Preoperative LVEF ≤0.45	2 (4)
Mitral valve pathology^a^	
Prolapse ≥2 anterior leaflet scallops	22 (39)
Prolapse ≥2 posterior leaflet scallops	30 (54)
Mitral repair procedures	
Annuloplasty	
Future Annuloplasty band^b^	8 (14)
Duran Ancore^b^	1 (2)
Profile 3D^b^	2 (4)
Carpentier-Edwards physio ring II^c^	42 (75)
Cosgrove annuloplasty^c^	2 (4)
Posterior leaflet resection	
Triangular	15 (27)
Quadrangular with sliding plasty	12 (21)
Artificial neochordae	52 (93)
Leaflet plication	7 (13)
Concomitant procedures	
Aortic valve replacement	2 (4)
Coronary artery bypass grafting	6 (11)
Maze procedure	25 (45)
Tricuspid ring annuloplasty	28 (50)

^a^Based on surgical assessment of the valve leaflets. ^b^Medtronic, Minneapolis, MN. ^c^Edwards Lifesciences, Irvine, CA. *LVEF* left ventricle ejection fraction

A mitral annuloplasty was added to the procedure for all patients except for 1 patient who underwent suture annuloplasty due to severe annular calcification. Annuloplasty was performed with the semirigid Future annuloplasty band (Medtronic, Minneapolis, MN) in 8 (14%) patients, Carpentier-Edwards Physio Ring II (Edwards Lifesciences, Irvine, CA) in 42 (75%), Cosgrove annuloplasty band (Edwards Lifesciences) in 2 (4%), Profile 3D (Medtronic, Minneapolis, MN) in 2 (4%), and with the Duran Ancore (Medtronic, Minneapolis, MN) in 1 (2%) patient (Table 1). The mean annuloplasty size was 30.6 ± 2.5 mm. Transesophageal echocardiography after weaning from cardiopulmonary bypass was performed routinely to control for the quality of the repair. If there was a residual MR greater than the trivial MR, then a second run of bypass was undertaken for correction. All patients received warfarin sodium postoperatively during the first 3 months for sinus rhythm and permanently if atrial fibrillation or flutter occurred.

### Statistical analyses

Data are presented as the means with standard deviations or median with interquartile range as appropriate. Freedom from recurrent MR of moderate or severe degree was calculated using Kaplan-Meier analysis. Repair durability was obtained by echocardiographic follow-up. Statistical analyses were performed using JMP software (version 14.1.0; SAS Institute, Cary, NC).

## Results

The general characteristics of the patients are described in Table 1. The patients with MR due to bileaflet lesions had a mean age of 67 ± 12 years and 19 (34%) were women. Prolapse of 2 or more anterior leaflet scallops and posterior leaflet scallops were observed in 22 (39%) and 30 (54%) patients, respectively. Concomitant to mitral repair, a maze procedure was performed in 25 (45%), coronary artery bypass grafting in 6 (11%), tricuspid ring annuloplasty in 28 (50%), and aortic valve replacement in 2 (4%) patients.

### Recurrent mitral regurgitation

**Fig. 1:**
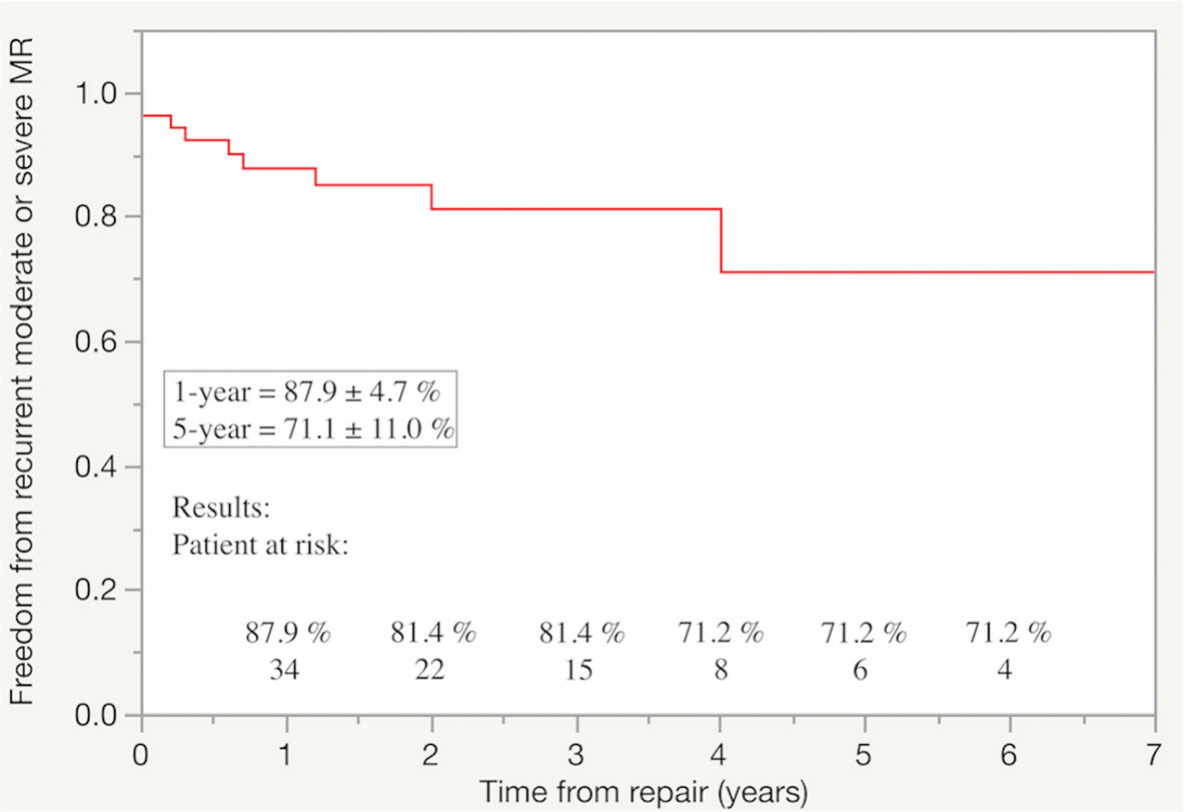
Freedom from recurrent moderate or severe mitral regurgitation (MR) after mitral valve (MV) repair of bileaflet lesions are shown. At follow-up, 10 patients had recurrent moderate or severe MR

Residual MR with mild or higher and systolic anterior motion of the MV was observed in 5 (9%) patients at the intraoperative post-repair transesophageal echocardiography. Of these, 3 patients underwent a second cardiac arrest to repair the residual MR with additional leaflet approximation or artificial neochordae placement. Height reduction with P2 resection was performed to repair systolic anterior motion in 2 other patients. Eleven (19.6%) and 2 (3.5%) patients had mild and moderate MR, respectively, on the first transthoracic echocardiogram 1 week after the repair; the remaining patients had no or trivial MR. Of the 2 patients with moderate MR, 1 patient had recurrent moderate MR due to failure of the artificial neochordae to the anterior leaflet and subsequently underwent successful re-repair with new artificial neochordae. The other patient had moderate MR due to anterior mitral leaflet tethering and decreased mitral leaflet motion. She was managed with medical therapy and echocardiography before discharge revealed mild MR. During the follow-up period, 10 (17.8%) patients developed moderate or severe MR. Freedom from recurrent moderate or severe MR was 87.9 ± 4.7% at 1 year and 71.1 ± 11.0% at 5 years (Fig. 1).

**Fig. 2:**
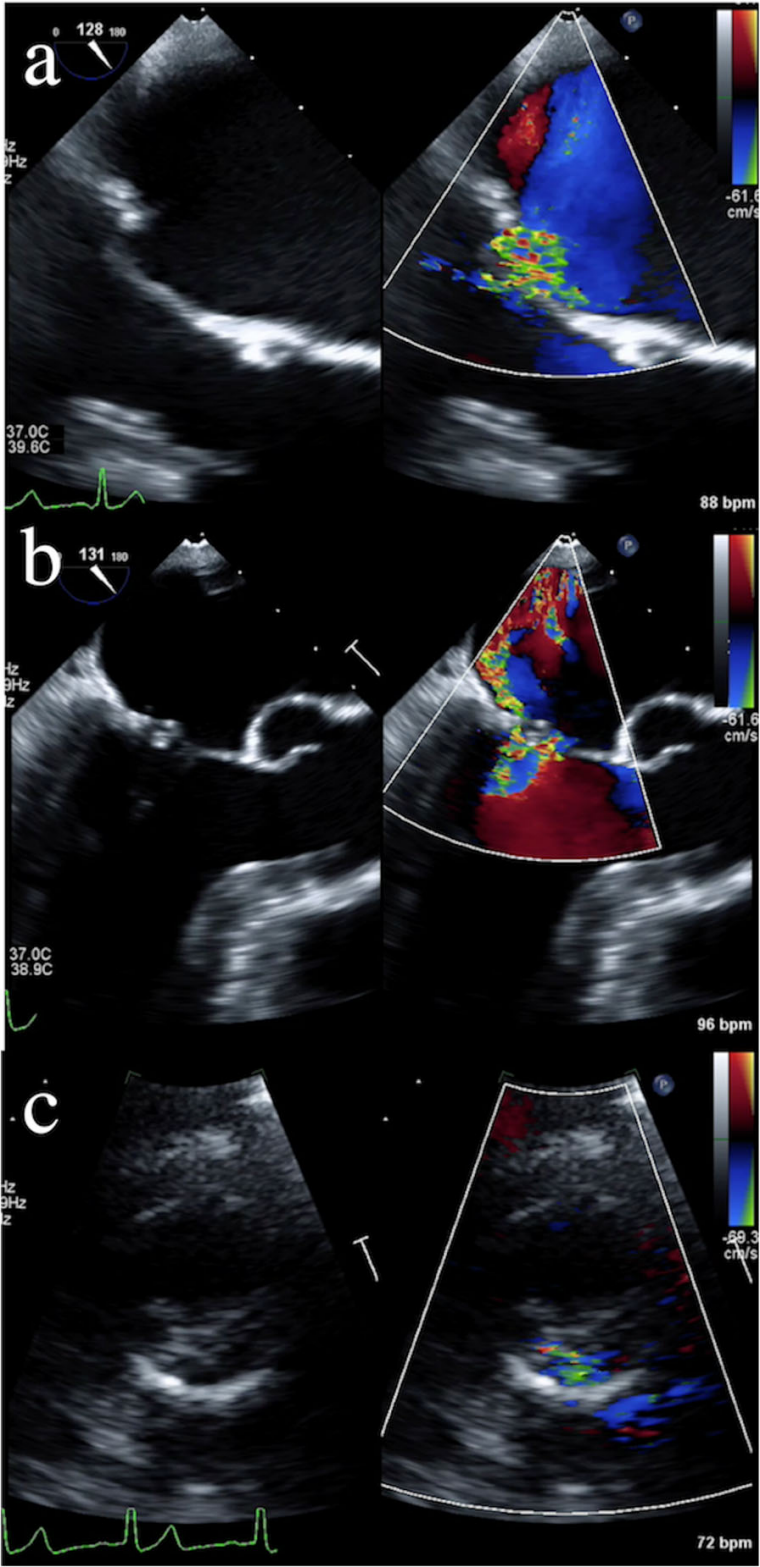
**a** Transesophageal echocardiography 8 days (early) after repair with neochordoplasty of A2 and quadrangular resection with sliding plasty of P2 showing anterior leaflet tethering and decreased motion of the posterior leaflet. **b** Transesophageal echocardiography 7 days (early) after repair with neochordoplasty of A2–3 showing A3 prolapse. **c** Transthoracic echocardiography 7 years (late) after repair with neochordoplasty of A2–3 and commissural plication of the posteromedial commissure showing a recurrent regurgitant jet at the posteromedial commissure

The interval from MV repair to the development of recurrent MR was a mean of 1.8 ± 2.1 years. The timing of recurrent MR was categorized into less than 1 year after the initial repair (early, median 91 days; *n* = 6) and more than 1 year (late, median 3.0 years; *n* = 4) (Table 2). Early recurrent MR was categorized by etiology into anterior leaflet tethering (*n* = 3) and prolapse (*n* = 3) after artificial neochordal placement. Late recurrent MR was mainly due to an MR jet from the anterior or posterior commissural area that was repaired for commissural prolapse (Fig. 2, Table 2).

**Table 2:** Etiology of recurrent moderate or severe mitral regurgitation after mitral valve repair

No.	Age/sex	MR lesion^a^	Repair technique	Interval from repair (years)	Etiology of failure^b^
1	70/M	A2–3 + P1–2 prolapse	Artificial neochordae of A2–3	0.0 (7 days)	A3 prolapse (failure of neochordae)
2	70/F	A2 + P2–3 prolapse	Quadrangular resection of P2–3, Artificial neochordae of A2	0.0 (8 days)	AML tethering, PML motion↓
3	60/M	A2 + P2-P3-PC prolapse	Artificial neochordae of A2, Quadrangular resection of P2, Commissural plication of PC	0.2	A2 prolapse (failure of neochordae)
4	77/F	A2 + P2 prolapse	Artificial neochordae of A2 + P2	0.3	P2 prolapse (failure of neochordae)
5	69/M	A2 prolapse, P1 gap	Artificial neochordae of A2, Leaflet approximation of P1	0.6	AML + PML tethering
6	77/F	A2 prolapse, PML tethering	Artificial neochordae of A2, Commissural plication of PC	0.7	A2–3 tethering
7	80/F	A1 + A3 + P3 prolapse, MAC	Artificial neochordae of A1, Commissural plication of PC	1.2	P3 prolapse
8	70/F	A2–3 prolapse, P2–3 gap	Artificial chordae of A2–3, Commissural plication of PC, Leaflet approximation of P2–3	2.0	A3 prolapse
9	69/M	A1 prolapse, P1 tethering	Triangular resection + Artificial neochordae of A1, Commissural plication of AC	4.0	A1 prolapse
10	68/M	A2–3 + PC prolapse	Artificial neochordae of A2–3, Commissural plication of PC	7.0	Regurgitation from PC

^a^Based on surgical assessments of the valve leaflets. ^b^Based on postoperative transthoracic and transesophageal echocardiography performed after mitral valve repair

*AC* anterolateral commissure, *AML* anterior mitral leaflet, *MAC* mitral annular calcification, *MR* mitral regurgitation, *PC* posteromedial commissure, *PML* posterior mitral leaflet

Among 10 patients with moderate or severe MR, 3 had reoperations, 6 were alive (4 patients with moderate MR were asymptomatic with normal ventricular function without reoperation), and 4 died (2 valve- or cardiac-related deaths and 2 noncardiac deaths).

### Reoperation on the mitral valve

Three patients underwent repeat MV surgery. The first patient had recurrent moderate MR without symptoms; however, reoperation was performed with Valsalva aneurysmal repair 5.2 years after the initial repair. On surgical inspection, A1 prolapse and P1 tethering, which were the same lesions necessitating the initial repair, were observed. Although the patient underwent re-repair with commissural plication, he had re-recurrent moderate MR 1 year after the reoperation and died 1.9 years after the re-repair due to congestive heart failure. The second patient had recurrent moderate MR without symptoms 3 months after the initial repair. P2 prolapse was suspected due to elongation of the artificial neochordae on postoperative transthoracic echocardiography at that time. Although the patient was initially followed up with medical therapy, she developed symptoms of congestive heart failure and underwent reoperation after 2.6 years. Reoperation revealed elongation of the artificial chordae to P2 and thickening of the posterior mitral leaflet. She underwent MV replacement with bioprosthesis since re-repair could not be performed due to progressive degeneration of the posterior mitral leaflet. The last patient had recurrent moderate MR with NYHA III symptoms 7 days after the initial repair due to failure of the artificial neochordae to the anterior leaflet. These chordae were found to be elongated on reoperation. He subsequently underwent successful re-repair with new artificial neochordae (Table 3).

**Table 3:** Reoperation for recurrent severe mitral regurgitation after mitral valve repair

No.	Age/sex	Repair technique at initial repair	Redo-operative findings	Redo-operative procedures
1	70/M	Artificial neochordae of A2–3	Elongation of artificial neochordae of A2–3.	Re-repair with artificial neochordae of A2–3.
4	77/F	Artificial neochordae of A2 + P2	Elongation of artificial neochordae to P2, PML thickening.	Re-replacement with bioprosthesis.
9	69/M	Triangular resection + Artificial neochordae of A1, Commissural plication of AC	A1 prolapse, P1 tethering.	Re-repair with commissural plication of AC.

*AC* anterolateral commissure, *PML* posterior mitral leaflet

Cardiac- and valve-related complications were observed in 18 patients. Nine patients experienced at least 1 episode of postoperative paroxysmal atrial fibrillation. One patient with recurrent moderate MR had an episode of infective endocarditis, which was treated with antibiotics alone. One patient on warfarin sodium for atrial fibrillation experienced gastrointestinal bleeding. There were no early (< 31 days) deaths, but 5 late deaths (3 cardiac- or valve-related and 2 other causes) occurred among 56 patients. The causes of the late cardiac- and valve-related deaths were congestive heart failure in 3 patients, intracranial bleeding in 1 patient on warfarin for atrial fibrillation, and septic shock due to acute cholecystitis in 1 patient.

## Discussion

This study provides information on mid-term clinical and echocardiographic outcomes with detailed mechanisms of recurrent MR focusing on bileaflet MV repair due to degenerative disease. Whereas posterior leaflet prolapse now can be repaired in practically all cases, with very low morbidity and excellent outcomes, many published series have documented decreased repair rates in the setting of anterior and bileaflet lesions [1–6].

The long-term durability of MV repair of bileaflet lesions is variable even among experienced or high-volume centers [2, 12–14]. In the present study, the freedom from recurrent moderate or severe MR was 87.9 ± 4.7% at 1 year and 71.1 ± 11.0% at 5 years. Although these results were inferior to the results from other centers, the high ratio of artificial neochordae placement and exclusion of limited commissural lesions in our cohort might reflect more complex MV pathology. The present study showed that 3 out of 10 patients with recurrent MR finally died of congestive heart failure. Gillinov et al. [15] reported that when a valve either appears unrepairable or attempts to repair fail because of complex valve pathology, neither survival nor reoperation is adversely affected by replacement. Therefore, we should know what types of MV lesions and MV repair techniques are associated with recurrent MR in complex valve pathology.

Some authors have demonstrated that the timing of failed repair can be categorized as early for procedure-related failure and later for valve-related failure, as in the present study [4, 16]. One of the challenges in categorizing late failure after the repair pertains to distinguishing between recurrent MR caused by a technical failure during surgery versus recurrent valve leakage caused by progression of native disease [17]. However, when recurrent MR occurs late after the repair of degenerative MV disease, new valve pathology is usually the culprit and re-repair is less common. In contrast, reoperation for procedure-related failure occurs early and is often amenable to re-repair [4, 16]. Indeed, early recurrent MR due to inappropriate artificial neochordae length, which was an etiology of early recurrent MR in the present study, was successfully re-repaired. Conversely, re-repair performed on the patient with recurrent MR occurring late after commissural repair resulted in re-recurrent MR. Suri et al. demonstrated that repair is clearly beneficial, conveying improved survival with better recovery of left ventricular function and left ventricular regression compared with valve replacement [3]. Thus, patients should be operated on at an early phase (asymptomatic or mildly symptomatic) because there is a higher probability of repair and a greater benefit on long-term survival [18]. We experienced 1 patient who required valve replacement due to posterior leaflet degeneration 2.6 years after MV repair. Although the patient did not have any symptoms with recurrent moderate MR, we should have performed re-repair before the MR jet caused progressive degeneration of MV leaflets.

Early failure can be caused by failure of neochordoplasty that results in increased strain on remaining chords or aggressive leaflet resection that results in late fibrosis and decreased mobility [17]. One of the challenges in MV repair for the treatment of bileaflet lesions is that neochordoplasty and leaflet resection are required on the anterior and posterior mitral leaflets, respectively. Unlike single anterior or posterior mitral leaflet repair, it is difficult to decide the area of resection or length of artificial neochordae to achieve sufficient coaptation length because the geometry may be changed after every step of repair. Quadrangular resection may preserve less valve function and leaflet kinematics than triangular resection or a non-resection technique [19]. Adjustment of the length of the artificial neochordae is sensitive, and inappropriate length can easily cause recurrent MR. Furthermore, Gillinov et al. reported that 70% of patients who demonstrated bileaflet prolapse on echocardiography did not have any significant anterior chordal pathology [11]. This means that bileaflet prolapse can be repaired only by posterior leaflet repair, and there is a risk of performing unnecessary neochordoplasty by wrong evaluations of the MV lesions. Considering the experiences of failure in MV repair in the present study, we suggest the following sequential approaches, in accordance with the suggestions by Castillo et al. [12] Repair of the posterior leaflet must be performed first by triangular resection or neochordoplasty to avoid aggressive leaflet resection. Next, whether anterior leaflet repair is needed must be re-evaluated based on the status of the chordae to the anterior leaflet [11]. Thereafter, if neochordoplasty is required to repair the anterior mitral leaflet lesions, re-evaluation can be performed to decide if additional width or height resection of the posterior MV leaflets is required after anterior MV repair. Lastly, fine-tuning of the artificial neochordae is required.

The number of studies pertaining to the long-term outcomes after MV repair for commissural lesions is limited. Shimizu et al. reviewed 122 patients with isolated commissural prolapse, which was repaired with leaflet resection or chordal replacement [10]. They reported that freedom from recurrent moderate or severe MR at 15 years was 87.4%. De Bonis et al. also reported the long-term outcomes of commissural plication with mitral annuloplasty for isolated commissural prolapse [9]. In their study, the freedom from moderate-severe or severe MR at 11 years was 96.3 ± 1.7%. However, if moderate MR was taken into consideration, 13 of 121 (10.7%) patients had recurrent MR. Therefore, more than one-tenth of patients who underwent MV repair for commissural lesions can develop recurrent MR during the long-term follow-up period. The mid-term outcomes of MV repair for commissural prolapse with plication and neochordoplasty in the present study were also unsatisfactory despite the lack of recurrent MR in the early phase. Although this implies that there may be underlying unknown failure mechanisms in commissural repair, the present study could not reveal the risk factors or specific mechanisms owing to the small number of samples. Further studies are warranted to elucidate the mechanisms of failure and explore better repair techniques for commissural lesions.

### Limitations

This study has the inherent limitations of any observational study. Several surgeons performed the surgical procedures and the results may have been affected by each surgeon’s skill. Despite the high proportion of patient follow-up, bias may have occurred among patients presenting for follow-up assessment and those who did not. MV function was assessed in multiple echocardiography laboratories and the interpretation of the results may not have been consistent. In addition, the mechanisms of recurrent MR were mainly evaluated on transthoracic echocardiography. Thus, further studies with evaluation on transesophageal echocardiography or surgical inspection during reoperation will be required to reveal more detailed mechanisms of recurrent MR.

## Conclusions

In the present study, 17% of patients developed significant recurrent MR at some point despite multiple reconstructive techniques. Aggressive resection of the posterior mitral leaflet or maladjustment of the artificial neochordae was considered as the mechanism of repair failure in the early phase. Repair failure can be avoided by using a sequential approach in which the posterior mitral leaflet is repaired first with limited resection area, followed by assessment and repair of the anterior mitral leaflet with neochordoplasty. Recurrent MR might occur in the late phase even after satisfactory commissural repair.

## Data Availability

The datasets used and analyzed during the current study are available from the corresponding author on reasonable request.

## Abbreviations

MR: mitral regurgitation
MV: mitral valve
